# Supplementary material for: Extracellular vesicles from symbiotic vaginal lactobacilli inhibit HIV-1 infection of human tissues
Source: Nat Commun. 2019 Dec 11;10:5656. doi: 10.1038/s41467-019-13468-9 (PMC6906448; doi:10.1038/s41467-019-13468-9)
Supplement: Supplementary file 1 — Supplementary Information [file 41467_2019_13468_MOESM1_ESM.pdf]

Supplementary Information

EXTRACELLULAR VESICLES FROM SYMBIOTIC VAGINAL LACTOBACILLI INHIBIT HIV-1 INFECTION OF HUMAN TISSUES

Ñahui Palomino et al.

Supplementary Figure 1

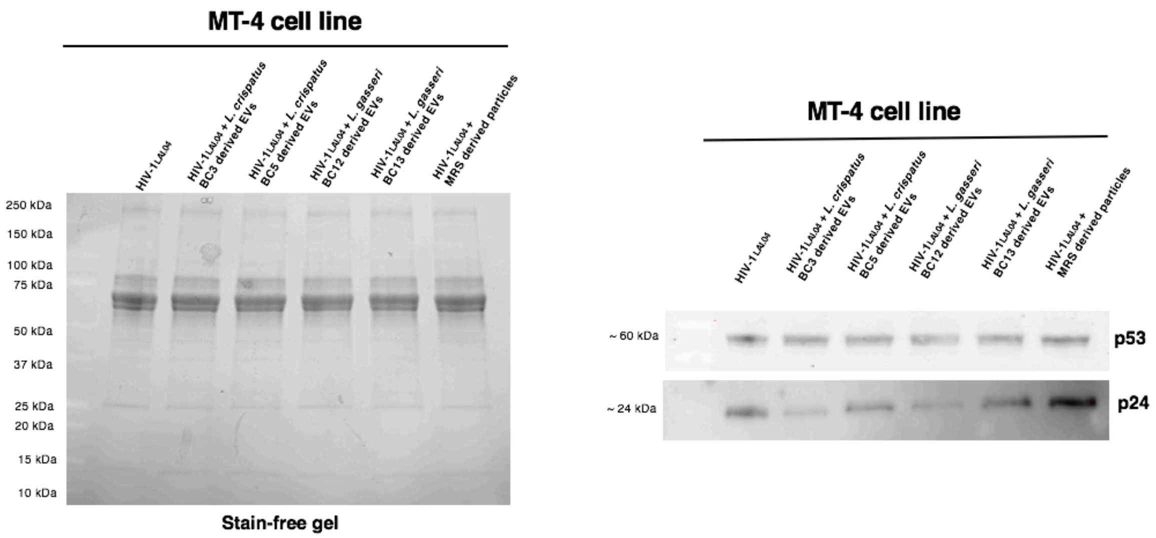

Supplementary Figure 1. Western blot of HIV-$1_{LAI.04}$ infected MT-4 cells after treatment or no treatment with bacterial EVs. Stain-free gel representing the spectrum of proteins obtained from cell lysates (right panel). Detection of p53 (~ 60 kDa) and p24 (~ 24 kDa) proteins (left panel). Source data are provided as a Source Data file

Supplementary Figure 2

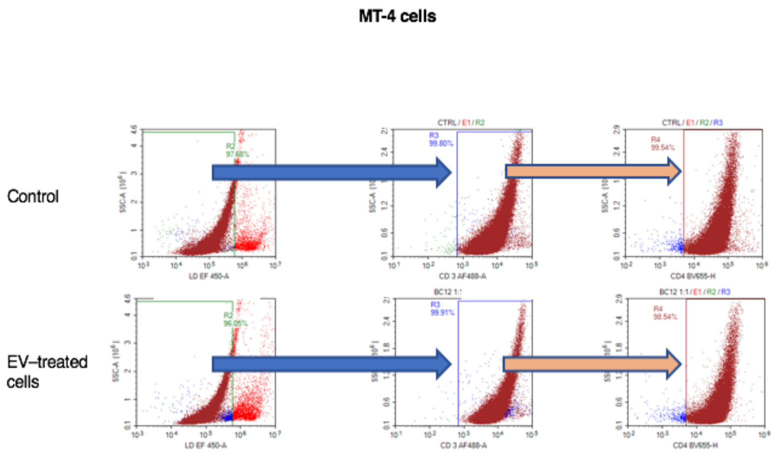

Supplementary Figure 2. Gating strategy for live/dead, CD3, and CD4 cells from MT-4 cells. Cell depletion in MT-4 cell cultures treated with *L. gasseri* BC12-derived EVs, as measured with flow cytometry. Panels from left to right represent staining for live/dead cells, CD3, and CD4 populations in EV-untreated cells (upper row) and in cells treated with *Lactobacillus*-derived EVs (lower row).

Supplementary Table 1. Metabolomic analysis of EVs derived from lactobacilli and particles derived from MRS

EV metabolite cargo	MRS derived EVs	*L. crispatus* BC3 derived EVs	*L. crispatus* BC5 derived EVs	*L. gasseri* BC12 derived EVs	*L. gasseri* BC13 derived EVs	r	*p*
**Organic acids**							
	1.90E-02	5.05E-02	3.49E-02	4.69E-02	3.95E-02		
1. Succinate	1.83E-02	4.97E-02	1.06E-01	5.17E-02	4.24E-02	0.26	0.352
	1.74E-02	5.02E-02	3.45E-02	5.05E-02	4.10E-02		
	3.43E-03	1.34E-02	1.07E-02	7.54E-03	7.95E-03		
2. Pyruvate	3.08E-03	1.20E-02	2.62E-02	6.87E-03	7.24E-03	0.03	0.920
	2.43E-03	1.05E-02	8.75E-03	7.22E-03	6.22E-03		
	1.91E-01	8.43E-01	1.44E+00	3.11E+00	1.98E+00		
3. Lactate	1.89E-01	8.37E-01	4.12E+00	3.14E+00	2.08E+00	0.38	0.168
	1.83E-01	8.00E-01	1.48E+00	3.18E+00	1.99E+00		
	3.13E-02	2.76E-02	2.72E-02	2.80E-02	2.67E-02		
4. Formate	2.81E-02	2.86E-02	3.57E-02	2.66E-02	2.87E-02	-0.32	0.244
	2.98E-02	2.94E-02	2.90E-02	2.91E-02	2.90E-02		
	2.48E+00	2.64E+00	2.21E+00	2.08E+00	2.42E+00		
5. Acetate	2.52E+00	2.62E+00	2.40E+00	2.10E+00	2.42E+00	-0.35	0.196
	2.38E+00	2.58E+00	2.29E+00	2.12E+00	2.42E+00		
**Amino acids and derivatives**							
	6.56E-02	7.46E-02	7.05E-02	8.69E-02	9.44E-02		
6. Valine	6.52E-02	7.45E-02	7.88E-02	8.83E-02	9.37E-02	0.33	0.224
	6.28E-02	7.32E-02	7.03E-02	9.01E-02	9.39E-02		
	2.87E-02	2.49E-02	2.55E-02	2.65E-02	2.34E-02		
7. Tryptophan	2.82E-02	2.33E-02	2.07E-02	2.37E-02	2.00E-02	-0.10	0.722
	2.67E-02	2.26E-02	2.30E-02	2.10E-02	2.04E-02		
	1.29E-01	1.13E-01	9.64E-02	1.22E-01	1.16E-01		
8. Threonine	1.22E-01	1.13E-01	9.22E-02	1.16E-01	1.14E-01	0.28	0.303
	1.17E-01	1.09E-01	1.02E-01	1.24E-01	1.17E-01		

	8.30E-02	8.89E-02	8.39E-02	8.76E-02	9.25E-02		
9. Tyrosine	9.04E-02	8.84E-02	9.24E-02	8.46E-02	9.28E-02	-0.22	0.428
	8.34E-02	8.60E-02	9.08E-02	8.92E-02	9.50E-02		
	1.14E-01	1.15E-01	1.07E-01	1.04E-01	9.81E-02		
10. Phenylalanine	1.19E-01	1.18E-01	1.05E-01	1.06E-01	1.03E-01	0.03	0.906
	1.13E-01	1.16E-01	1.10E-01	1.05E-01	1.01E-01		
	1.62E-01	1.80E-01	1.60E-01	1.68E-01	1.82E-01		
11. Leucine	1.64E-01	1.80E-01	1.76E-01	1.70E-01	1.84E-01	0.18	0.531
	1.56E-01	1.76E-01	1.64E-01	1.73E-01	1.84E-01		
	5.33E-02	5.98E-02	5.83E-02	6.52E-02	6.66E-02		
12. Isoleucine	5.23E-02	5.90E-02	5.92E-02	6.71E-02	6.71E-02	0.49	0.067
	4.92E-02	5.83E-02	5.78E-02	6.91E-02	6.72E-02		
	**1.16E-01**	**1.18E-01**	**1.10E-01**	**1.46E-01**	**1.32E-01**		
**13. Glutamate**	**1.23E-01**	**1.16E-01**	**1.15E-01**	**1.41E-01**	**1.31E-01**	**0.61**	**0.016**
	**1.12E-01**	**1.12E-01**	**1.08E-01**	**1.50E-01**	**1.29E-01**		
	**8.79E-02**	**9.89E-02**	**8.33E-02**	**1.10E-01**	**1.04E-01**		
**14. Glycine**	**8.83E-02**	**9.69E-02**	**9.39E-02**	**1.11E-01**	**1.04E-01**	**0.72**	**0.002**
	**8.56E-02**	**9.72E-02**	**8.49E-02**	**1.14E-01**	**1.04E-01**		
	3.14E-02	2.99E-02	3.22E-02	3.34E-02	2.21E-02		
15. Aspartate	3.72E-02	3.17E-02	3.13E-02	3.54E-02	2.22E-02	0.43	0.106
	3.25E-02	2.94E-02	3.36E-02	4.13E-02	2.34E-02		
	1.71E-01	1.91E-01	1.70E-01	1.82E-01	2.11E-01		
16. Alanine	1.75E-01	1.91E-01	1.51E-01	1.85E-01	2.13E-01	0.16	0.560
	1.62E-01	1.86E-01	1.76E-01	1.92E-01	2.15E-01		
	**4.87E-02**	**3.27E-02**	**3.29E-02**	**3.05E-02**	**4.05E-02**		
**17. Asparagine**	**4.73E-02**	**3.41E-02**	**4.24E-02**	**2.77E-02**	**4.45E-02**	**-0.81**	**0.000**
	**4.60E-02**	**2.95E-02**	**4.26E-02**	**3.37E-02**	**3.76E-02**		
	6.69E-02	5.97E-02	6.25E-02	5.96E-02	5.73E-02		
18. Lysine	6.86E-02	5.86E-02	6.08E-02	5.94E-02	5.99E-02	-0.29	0.294
	6.39E-02	6.16E-02	5.82E-02	6.29E-02	6.06E-02		
**19. Methionine**	**3.38E-02**	**3.58E-02**	**3.62E-02**	**3.66E-02**	**3.51E-02**	**0.62**	**0.014**
	**3.34E-02**	**3.63E-02**	**3.59E-02**	**3.76E-02**	**3.62E-02**		

	**3.34E-02**	**3.51E-02**	**3.37E-02**	**3.59E-02**	**3.60E-02**		
	4.08E-03	3.36E-03	3.92E-03	4.03E-03	3.62E-03		
20. Creatine	4.38E-03	3.40E-03	4.61E-03	3.71E-03	3.49E-03	-0.23	0.413
	3.93E-03	3.39E-03	3.48E-03	4.04E-03	3.70E-03		
	9.05E-02	8.87E-02	8.16E-02	9.69E-02	1.18E-01		
21. Pyroglutamate	8.19E-02	8.35E-02	9.49E-02	1.09E-01	1.17E-01	0.18	0.528
	7.96E-02	8.70E-02	7.39E-02	1.10E-01	1.18E-01		
**Sugars**							
	5.29E+00	4.35E+00	3.58E+00	1.46E+00	1.65E+00		
22. Glucose	5.35E+00	4.37E+00	5.97E-01	1.47E+00	1.65E+00	-0.27	0.334
	5.09E+00	4.24E+00	3.71E+00	1.51E+00	1.63E+00		
	5.18E-02	5.14E-02	4.66E-02	3.97E-02	4.72E-02		
23. Trehalose	5.41E-02	4.98E-02	3.99E-02	4.01E-02	4.66E-02	-0.44	0.098
	4.99E-02	5.05E-02	4.59E-02	4.05E-02	4.59E-02		
	1.39E-03	8.99E-03	3.25E-02	6.55E-03	1.35E-02		
24. Galactose	1.51E-02	6.25E-03	9.71E-03	8.42E-03	1.36E-02	-0.40	0.139
	1.16E-02	8.91E-03	1.27E-02	7.08E-03	9.57E-03		
	2.36E-01	2.22E-01	1.82E-01	1.97E-01	2.25E-01		
25. Lactose	2.16E-01	2.10E-01	2.08E-01	2.04E-01	2.19E-01	-0.10	0.712
	1.98E-01	2.13E-01	1.92E-01	2.11E-01	2.22E-01		
**Nitrogen bases and analogs**							
	1.30E-02	7.20E-03	9.42E-03	1.83E-02	6.33E-03		
26. Uracil	9.98E-03	7.72E-03	7.94E-03	1.39E-02	6.03E-03	0.49	0.062
	1.07E-02	6.54E-03	1.15E-02	1.45E-02	6.92E-03		
	7.88E-03	9.44E-03	9.63E-03	1.30E-02	5.86E-03		
27. Cytosine	8.58E-03	9.47E-03	1.27E-02	1.18E-02	7.85E-03	0.47	0.080
	8.90E-03	7.98E-03	1.25E-02	1.19E-02	6.63E-03		
	**7.63E-03**	**8.19E-03**	**1.40E-02**	**1.86E-02**	**8.51E-03**		
**28. Hypoxanthine**	**8.47E-03**	**9.96E-03**	**1.28E-02**	**1.92E-02**	**9.16E-03**	**0.68**	**0.005**
	**8.22E-03**	**1.01E-02**	**1.35E-02**	**1.98E-02**	**8.38E-03**		
29. Uridine	7.92E-03	6.79E-03	9.37E-03	5.85E-03	2.32E-03	-0.27	0.338

	9.48E-03	7.21E-03	7.83E-03	3.81E-03	2.49E-03		
	9.07E-03	6.54E-03	1.08E-02	4.77E-03	1.51E-03		
	6.47E-03	8.61E-03	9.28E-03	7.45E-03	6.13E-03		
30. Adenine	6.12E-03	9.00E-03	4.28E-03	6.44E-03	6.09E-03	0.25	0.373
	4.27E-03	8.06E-03	9.72E-03	6.58E-03	6.35E-03		
**Others**							
	9.72E-03	1.02E-02	8.71E-03	8.66E-03	1.02E-02		
31. Choline	9.84E-03	1.03E-02	9.22E-03	8.78E-03	1.02E-02	-0.27	0.325
	9.42E-03	1.02E-02	8.93E-03	8.86E-03	1.02E-02		
	9.88E-03	1.03E-02	8.09E-03	7.20E-03	8.68E-03		
32. sn-Glycero-3-phosphocholine	1.01E-02	1.01E-02	1.06E-02	7.05E-03	8.24E-03	-0.40	0.139
	9.43E-03	9.89E-03	8.65E-03	7.58E-03	8.14E-03		
	4.75E-03	4.49E-03	5.59E-03	6.15E-03	4.97E-03		
33. Sarcosine	4.68E-03	4.50E-03	4.57E-03	5.57E-03	4.87E-03	0.36	0.192
	5.14E-03	4.13E-03	5.37E-03	6.02E-03	5.13E-03		
	5.82E-03	8.36E-03	7.18E-03	1.54E-02	1.61E-02		
34. 1,3-Dihydroxyacetone	6.00E-03	8.64E-03	1.29E-02	1.59E-02	1.58E-02	0.38	0.161
	5.18E-03	7.92E-03	6.35E-03	1.48E-02	1.53E-02		
	1.10E-02	9.36E-03	1.01E-02	7.83E-03	9.01E-03		
35. Hydroxyacetone	1.15E-02	9.43E-03	7.51E-03	8.98E-03	9.20E-03	-0.49	0.066
	1.00E-02	9.58E-03	1.13E-02	8.57E-03	9.13E-03		
	2.08E-02	2.97E-02	1.81E-02	2.10E-02	2.73E-02		
36. 4-Aminobutyrate	1.88E-02	2.68E-02	2.92E-02	1.98E-02	2.50E-02	0.04	0.879
	1.57E-02	2.18E-02	1.83E-02	1.85E-02	2.12E-02		
	4.32E-03	4.69E-03	4.06E-03	5.33E-03	3.37E-03		
37. Histamine	3.81E-03	4.41E-03	6.87E-03	4.07E-03	3.14E-03	0.36	0.187
	3.76E-03	5.15E-03	4.33E-03	5.33E-03	3.15E-03		
	3.65E-02	4.04E-02	4.58E-02	4.09E-02	4.09E-02		
38. myo-Inositol	4.61E-02	3.67E-02	3.15E-02	3.96E-02	4.20E-02	-0.25	0.374
	4.32E-02	3.86E-02	4.30E-02	3.69E-02	3.86E-02		
39. Glycerol	1.80E-02	4.01E-02	3.44E-02	3.79E-02	4.72E-02	0.37	0.175
	1.82E-02	3.89E-02	2.51E-02	3.75E-02	4.60E-02		

	1.69E-02	3.97E-02	3.35E-02	3.85E-02	4.60E-02		
	1.56E-01	1.20E-01	5.61E-02	1.15E-01	2.44E-02		
40. Ethanol	1.62E-01	1.24E-01	8.27E-02	1.18E-01	2.44E-02	0.31	0.266
	1.55E-01	1.17E-01	5.61E-02	1.16E-01	2.46E-02		
	1.25E-03	2.89E-03	3.59E-03	6.74E-04	8.43E-04		
41. Acetone	1.55E-03	2.81E-03	1.46E-03	9.05E-04	7.45E-04	-0.12	0.674
	1.08E-03	2.71E-03	3.38E-03	6.91E-04	7.52E-04		
	6.40E-03	7.04E-03	6.63E-03	5.50E-03	7.17E-03		
42. 2-Oxoglutarate	6.90E-03	6.93E-03	1.25E-02	6.08E-03	6.64E-03	-0.48	0.071
	7.05E-03	6.62E-03	7.15E-03	4.46E-03	6.98E-03		

Molecules significantly (p<0.05) related to antiviral activity were highlighted in **bold**. r, Pearson correlation coefficient.

Supplementary Table 2. Protein cargo of EVs derived from *L. gasseri* BC12 and *L. crispatus* BC5

***Lactobacillus*-EV associated identified proteins**	**Accession Number**	**Molecular Weight**	**GO terms: Cellular localization**	**GO terms: Molecular function**	***L. gasseri* BC12 derived EVs Run #1**	***L. gasseri* BC12 derived EVs Run #2**	***L. crispatus* BC5 derived EVs Run #1**	***L. crispatus* BC5 derived EVs Run #2**
1. ATP synthase subunit beta [*L. gasseri* ATCC 33323]	ATPB_LACGA	52 kDa	Membrane	ATP binding, proton-transporting ATP synthase activity, rotational mechanism	**15**	**11**	**3**	**2**
2. ATP synthase subunit alpha [*L. gasseri* ATCC 33323]	ATPA_LACGA (+1)	55 kDa	Membrane	ATP binding, proton-transporting ATP synthase activity, rotational mechanism	**9**	**9**	**1**	
3. Phosphonates import ATP-binding protein PhnC [*L. gasseri* ATCC 33323]	PHNC_LACGA	29 kDa	Membrane	ATP binding, ATPase-coupled organic phosphonate transmembrane transporter activity	**5**	**3**	**2**	**1**
4. ATP synthase subunit b [*L. gasseri* ATCC 33323]	ATPF_LACGA	18 kDa	Membrane	Proton-transporting ATP synthase activity, rotational mechanism	**7**	**5**	**1**	
5. Enolase 2 [*L. gasseri* ATCC 33323]	ENO2_LACGA	47 kDa	Cytoplasm Extracellular	Magnesium ion binding, phosphopyruvate hydratase activity	**4**	**5**		
6. 60 kDa chaperonin [*L. gasseri* ATCC 33323]	CH60_LACGA (+1)	58 kDa	Cytoplasm	ATP binding, unfolded protein binding	**3**	**4**		
7. Enolase 1 [*L. gasseri* ATCC 33323]	ENO1_LACGA (+1)	47 kDa	Cytoplasm Extracellular	Magnesium ion binding, phosphopyruvate hydratase activity	**4**	**3**	**3**	**3**
8. Elongation factor Tu [*L. gasseri* ATCC 33323]	EFTU_LACGA	44 kDa	Cytoplasm	GTP binding, GTPase activity, translation elongation factor activity	**7**	**5**		
9. ATP synthase gamma chain [*L. gasseri* ATCC 33323]	ATPG_LACGA	35 kDa	Membrane	ATP binding, proton-transporting ATP synthase activity, rotational mechanism	**6**	**5**		
10. Foldase protein PrsA 1 [*L. johnsonii* NCC 533]	PRSA1_LACJO	33 kDa	Membrane	Peptidyl-prolyl cis-trans isomerase activity	**5**	**4**		
11. ATP synthase subunit beta [*L. acidophilus* ATCC 700396]	ATPB_LACAC	52 kDa	Membrane	ATP binding, proton-transporting ATP synthase activity, rotational mechanism	**1**	**1**	**2**	**3**
12. ATP synthase subunit delta [*L. gasseri* ATCC 33323]	ATPD_LACGA	21 kDa	Membrane	Proton-transporting ATP synthase activity, rotational mechanism	**2**	**1**		
13. 50S ribosomal protein L4 [*L. acidophilus* ATCC 700396]	RL4_LACAC	22 kDa	Cytoplasm	rRNA binding			**2**	
14. Pyruvate kinase [*L. delbrueckii* subsp. Bulgaricus]	KPYK_LACDE	63 kDa	Cytoplasm	ATP binding, kinase activity, magnesium ion binding, potassium ion binding, pyruvate kinase activity	**3**			
15. 30S ribosomal protein S4 [*L. gasseri* ATCC 33323]	RS4_LACGA (+1)	23 kDa	Cytoplasm	rRNA binding		**1**	**2**	**1**
16. Triosephosphate isomerase [*L. gasseri* ATCC 33323]	TPIS_LACGA (+1)	27 kDa	Cytoplasm	Triose-phosphate isomerase activity	**2**	**1**		
17. 50S ribosomal protein L21 [*L. acidophilus* ATCC 700396]	RL21_LACAC (+1)	11 kDa	Cytoplasm	rRNA binding			**2**	
18. 50S ribosomal protein L2 [*L. gasseri* ATCC 33323]	RL2_LACGA (+2)	30 kDa	Cytoplasm	rRNA binding, transferase activity			**2**	

In **bold** are shown the number of peptides with a probability greater than 95%.
