# Supplementary material for: Extracellular vesicles from symbiotic vaginal lactobacilli inhibit HIV-1 infection of human tissues
Source: Nat Commun. 2019 Dec 11;10:5656. doi: 10.1038/s41467-019-13468-9 (PMC6906448; doi:10.1038/s41467-019-13468-9)
Supplement: Supplementary file 3 — Reporting Summary [file 41467_2019_13468_MOESM3_ESM.pdf]

nature research

Corresponding author(s): Leonid Margolis

Last updated by author(s): Nov 5, 2019

Reporting Summary

Nature Research wishes to improve the reproducibility of the work that we publish. This form provides structure for consistency and transparency in reporting. For further information on Nature Research policies, see Authors & Referees and the Editorial Policy Checklist .

Statistics

For all statistical analyses, confirm that the following items are present in the figure legend, table legend, main text, or Methods section.

n/a	Confirmed	
☐	☒	The exact sample size (*n*) for each experimental group/condition, given as a discrete number and unit of measurement
☐	☒	A statement on whether measurements were taken from distinct samples or whether the same sample was measured repeatedly
☐	☒	The statistical test(s) used AND whether they are one- or two-sided<br>*Only common tests should be described solely by name; describe more complex techniques in the Methods section.*
☒	☐	A description of all covariates tested
☐	☒	A description of any assumptions or corrections, such as tests of normality and adjustment for multiple comparisons
☐	☒	A full description of the statistical parameters including central tendency (e.g. means) or other basic estimates (e.g. regression coefficient) AND variation (e.g. standard deviation) or associated estimates of uncertainty (e.g. confidence intervals)
☒	☐	For null hypothesis testing, the test statistic (e.g. *F*, *t*, *r*) with confidence intervals, effect sizes, degrees of freedom and *P* value noted<br>*Give P values as exact values whenever suitable.*
☒	☐	For Bayesian analysis, information on the choice of priors and Markov chain Monte Carlo settings
☒	☐	For hierarchical and complex designs, identification of the appropriate level for tests and full reporting of outcomes
☒	☐	Estimates of effect sizes (e.g. Cohen's *d*, Pearson's *r*), indicating how they were calculated

Our web collection on statistics for biologists contains articles on many of the points above.

Software and code

Policy information about availability of computer code

Data collection	NanoSight NS300 (Malvern instruments Ltd, Malvern, UK); Luminex 100 (Bio-Rad, Hercules, CA); Saphire 2 luminometer (Tecan, Switzerland); NucleoCounter NC-100 automated cell counting system (ChemoMetec, Denmark); Novocyte flow cytometer (ACEA Biosciences, CA); ChemiDoc (Bio-Rad, Hercules, CA); 1H-NMR spectra were recorded with an AVANCE III spectrometer (Bruker, Milan, Italy); LC-ESI-MS/MS were collected using 6560 IM Q-TOF mass spectrometer (Agilent Technologies, Santa Clara, CA).
Data analysis	NanoSight NTA sofware 3.0 (Malvern instruments Ltd, Malvern, UK); Luminex Bioplex manager software 4.1.1 (Bio-Rad, Hercules, CA); NovoExpress version 1.2.4 sofware (ACEA Biosciences, CA);The 1H-NMR signals were assigned by comparing their multiplicity and chemical shift with Chenomx software data bank (ver 8.1 Chenomx, Inc., Edmonton, Canada); Mascot version 2.6.2 (Matrix science, London, UK); Scaffold version 4.8.7 (Proteome Software Inc., Portland, OR); GraphPad prism version7, GraphPad prism software Inc., San Diego, CA; “corr.test” from the R.

For manuscripts utilizing custom algorithms or software that are central to the research but not yet described in published literature, software must be made available to editors/reviewers. We strongly encourage code deposition in a community repository (e.g. GitHub). See the Nature Research guidelines for submitting code & software for further information.

Data

Policy information about availability of data

All manuscripts must include a data availability statement. This statement should provide the following information, where applicable:

- Accession codes, unique identifiers, or web links for publicly available datasets
- A list of figures that have associated raw data
- A description of any restrictions on data availability

Associated raw data is provided as a Source Data file and is indicated in the in the appropriate figure legends.

Field-specific reporting

Please select the one below that is the best fit for your research. If you are not sure, read the appropriate sections before making your selection.

- [x] Life sciences
- [ ] Behavioural & social sciences
- [ ] Ecological, evolutionary & environmental sciences

For a reference copy of the document with all sections, see nature.com/documents/nr-reporting-summary-flat.pdf

Life sciences study design

All studies must disclose on these points even when the disclosure is negative.

Sample size	We performed experiments to have enough sample sizes to obtain reliable results. These sample sizes represent the standard practice for publication in this field. Each sample represents independent biological replicates. Statistical analysis for each experiment is reported in the results and figure legends.
Data exclusions	None
Replication	Each experiment was repeated at least three times, except for proteomics (n=2).
Randomization	Data collection and analysis were carried out on randomly selected samples.
Blinding	The investigators were blinded to group allocation during data collection and/or analyses.

Reporting for specific materials, systems and methods

We require information from authors about some types of materials, experimental systems and methods used in many studies. Here, indicate whether each material, system or method listed is relevant to your study. If you are not sure if a list item applies to your research, read the appropriate section before selecting a response.

Materials & experimental systems

n/a	Involved in the study	
☐	☒	Antibodies
☐	☒	Eukaryotic cell lines
☒	☐	Palaeontology
☒	☐	Animals and other organisms
☐	☒	Human research participants
☒	☐	Clinical data

Methods

n/a	Involved in the study	
☒	☐	ChIP-seq
☐	☒	Flow cytometry
☒	☐	MRI-based neuroimaging

Antibodies

Antibodies used	Anti-CD3-AF488 fluorescence-labeled monoclonal antibody (Thermo Fisher Scientific, Waltham, MA; catalog # MHCD0320);<br>Anti-CD4-BV605 fluorescence-labeled monoclonal antibody (BD Bioscences, San Jose, CA; catalog # 562658);<br>Human monoclonal PG9 antibody (Polymun Scientific, Austria; catalog # AB015);<br>Anti-HIV-1 p24 monoclonal antibody (Abcam, Cambridge, MA; catalog # ab9071);<br>Anti-p53 monoclonal antibody (Thermo Fisher Scientific, Waltham, MA; catalog # MA5-12557);<br>Anti-CD63 monoclonal antibody (Thermo Fisher Scientific, Waltham, MA; catalog # 10628D);<br>Anti-TSG101 monoclonal antibody (Thermo Fisher Scientific, Waltham, MA; catalog # MA1-23296);
Validation	Anti-CD3-AF488, specific for human CD3 delta;<br>Anti-CD4-BV605, specific for human CD4;<br>Human monoclonal PG9 antibody, specific for human structural epitope of HIV-1 gp120;<br>Anti-HIV-1 p24 monoclonal antibody, specific for human Immunodeficiency Virus Type 1(HIV 1) p24 protein;<br>Anti-p53 monoclonal antibody, specific for human and bovine p53;<br>Anti-CD63 monoclonal antibody, specific for human CD63;<br>Anti-TSG101 monoclonal antibody, specific for hamster, human, mouse, non-human primate, rat TSG101.

Eukaryotic cell lines

Policy information about cell lines

Cell line source(s)	Human T-lymphocyte MT-4 (obtained through the NIH AIDS Reagent Program, catalog number 120).<br>Jurkat-tat cell lines (obtained through the NIH AIDS Reagent Program, catalog number 1399).<br>TZM-bl cell line (obtained through the NIH AIDS Reagent Program,l catalog number 8129).
Authentication	MT-4, Jurkat-tat, and TZM-bl cell lines have been authenticated by NIH AIDS Reagent Program.
Mycoplasma contamination	Cell line cultures were free of mycoplasma contamination.
Commonly misidentified lines<br>(See ICLAC register)	No commonly misidentified cell lines were used.

Human research participants

Policy information about studies involving human research participants

Population characteristics	*Describe the covariate-relevant population characteristics of the human research participants (e.g. age, gender, genotypic information, past and current diagnosis and treatment categories). If you filled out the behavioural & social sciences study design questions and have nothing to add here, write "See above."*
Recruitment	Human tissues were obtained from routine surgery (unrelated to the current study) and according to all relevant ethical regulations for work with human participants including the patient's informed consent. Tissue samples were anonymized.
Ethics oversight	Tonsillectomies was performed in the Children's Hospital (Washington, DC). Tissues were received from the Pathology Department and were considered as "pathological waste". Tissue samples were anonymized and the protocol was approved by the Children's Hospital IRB and by the NIH Office of Human Subject Research. Cervico-vaginal tissues were received as anonymized samples from the National Disease Research Interchange (NDRI). NDRI maintains a Federal Wide Assurance (FWA00006180) agreement with the DHHS, Office for Human Research Protections to comply with federal regulations concerning research involving human subjects. NDRI's human tissue procurement programs and informed consent/ authorization documents were approved by the IRB#5 of the University of Pennsylvania.

Note that full information on the approval of the study protocol must also be provided in the manuscript.

Flow Cytometry

Plots

Confirm that:

- [x] The axis labels state the marker and fluorochrome used (e.g. CD4-FITC).
- [x] The axis scales are clearly visible. Include numbers along axes only for bottom left plot of group (a 'group' is an analysis of identical markers).
- [x] All plots are contour plots with outliers or pseudocolor plots.
- [x] A numerical value for number of cells or percentage (with statistics) is provided.

Methodology

Sample preparation	We evaluated cell depletion on MT-4 cells treated or not treated with bacterial EVs (derived from L. gasseri BC12). After 3 days of culture, cells were centrifuged at 400 × g, resuspended in staining buffer (PBS containing 2% mouse serum, 2% goat serum, and 2% FBS), and stained with live/dead Fixable Viability Dye eFluor 450 (ef 450, Invitrogen, Carlsbad, CA) for 20 minutes. After incubation, cells were washed and stained with anti-CD3-AF488 and anti-CD4-BV605 fluorescence-labeled monoclonal antibodies for 20 minutes.
Instrument	Data were acquired with a Novocyte flow cytometer (ACEA Biosciences, CA) equipped with 405, 488, and 650 nm laser lines.
Software	We analyzed the data using NovoExpress version 1.2.4 software (ACEA Biosciences, CA).
Cell population abundance	No sorts were performed for this study.
Gating strategy	First, we gated on the live population. In the live population, we next gated on CD3+ cells and then on CD4+ cells.

- [x] Tick this box to confirm that a figure exemplifying the gating strategy is provided in the Supplementary Information.
